# Epilepsy as a Multiscale Network Disorder: Integrating Precision Therapeutics and Emerging Experimental Platforms

**DOI:** 10.3390/pharmaceutics18080969

**Published:** 2026-08-07

**Authors:** Wonseok Chang, Amy Seomin Kwak, Seung Ho Han, Dae Yong Song, Hong Il Yoo, Jung Ho Lee

**Affiliations:** 1 Department of Physiology and Biophysics, Eulji University School of Medicine, Daejeon 34824, Republic of Korea; cws2017@eulji.ac.kr (W.C.); hans0424@eulji.ac.kr (S.H.H.); 2 Public Health Studies, Krieger School of Arts and Sciences, Johns Hopkins University, Baltimore, MD 21218, USA; skwak5@jh.edu; 3 Department of Anatomy and Neurosciences, Eulji University School of Medicine, Daejeon 34824, Republic of Korea; dysong@eulji.ac.kr; 4 Department of Biomedical Engineering, Johns Hopkins University School of Medicine, Baltimore, MD 21205, USA; 5 Department of Pharmacology, Eulji University School of Medicine, Daejeon 34824, Republic of Korea

**Keywords:** epilepsy, multiscale network, precision therapeutics, New Approach Methodologies (NAMs)

## Abstract

**Background/Objectives**: Epilepsy remains a major neurological disorder, with approximately one-third of patients continuing to experience pharmacoresistant seizures despite the availability of numerous antiseizure medications (ASMs). While current therapies primarily target neuronal hyperexcitability through modulation of ion channels and neurotransmitter systems, increasing evidence suggests that epileptogenesis arises from multiscale interactions involving molecular, cellular, circuit, network, neuroinflammatory, and neurovascular mechanisms. Although therapeutic strategies have diversified, this expanded mechanistic understanding has not yet been fully incorporated into therapeutic development and evaluation. This review integrates current knowledge of multiscale epilepsy pathophysiology with recent therapeutic advances and emerging experimental platforms. **Methods**: This narrative review synthesized literature identified primarily through PubMed and Google Scholar searches through January 2026, supplemented by targeted updates of therapeutic development and regulatory status through July 2026. Particular emphasis was placed on ion channel modulators, synaptic and neuromodulatory therapies, neuroinflammatory interventions, precision genetic approaches, and human-relevant experimental platforms, including induced pluripotent stem cell (iPSC)-derived models, brain organoids, multi-electrode arrays (MEAs), organ-on-a-chip systems, multi-omics technologies, and artificial intelligence (AI)-based analytical frameworks. **Results**: Current and emerging therapies target increasingly diverse molecular, circuit, neuromodulatory, and neuroinflammatory mechanisms. However, drug resistance remains multifactorial, and the long-term effects of therapeutic interventions on network remodeling, neuro-glial interactions, and sustained clinical response remain incompletely understood. NAMs provide complementary capabilities for patient-specific disease modeling, functional network phenotyping, neurovascular modeling, and the integration of molecular, electrophysiological, and computational data across biological scales. **Conclusions**: Epilepsy is increasingly recognized as a multiscale network disorder rather than solely a condition of neuronal hyperexcitability. The coordinated use of complementary human-relevant platforms may help incorporate multiscale mechanistic insights into therapeutic development and evaluation, narrow persistent translational gaps, and support more predictive and mechanism-informed treatment strategies.

## 1. Introduction

Epilepsy remains one of the most challenging neurological disorders despite the development of numerous antiseizure medications (ASMs), with nearly one-third of patients continuing to experience pharmacoresistant seizures [1]. Traditionally, epilepsy drug development has focused on suppressing neuronal hyperexcitability through modulation of ion channels and neurotransmitter systems. Although these approaches have improved seizure control, they have not fully addressed the complex and heterogeneous mechanisms underlying epileptogenesis or interindividual variability in treatment response.

Recent therapeutic strategies have increasingly shifted from the discovery of entirely new drug classes toward improving the efficacy, tolerability, and precision of existing therapies [2,3]. In particular, next-generation ASMs, targeted therapies for refractory and genetic epilepsies, and precision medicine approaches aimed at predicting patient-specific drug responses have gained growing attention [4]. The clinical expansion of cenobamate and the emergence of artificial intelligence (AI)-based platforms capable of predicting ASM responsiveness illustrate this transition toward personalized therapeutic optimization [5,6]. At the same time, novel approaches including gene-targeted therapies, RNA-based interventions, and digital therapeutics are broadening the therapeutic landscape beyond conventional seizure suppression [7,8].

Parallel advances in preclinical research have further emphasized the importance of network-level mechanisms in epilepsy. Contemporary studies increasingly investigate neuroinflammation, glial dysfunction, blood–brain barrier disruption, metabolic dysregulation, and pathological circuit synchronization as key contributors to epileptogenesis [9,10,11]. These developments support a growing conceptual shift from viewing epilepsy solely as a disorder of neuronal hyperexcitability toward understanding it as a multiscale network disorder involving interactions across molecular, cellular, circuit, and systems levels.

Although therapeutic strategies have evolved considerably, this expanded understanding of epilepsy pathophysiology has not yet been fully incorporated into the development, evaluation, and clinical application of emerging therapies. Therapeutic interventions may target specific molecular, cellular, or circuit-level mechanisms, yet their broader consequences within the epileptic network remain incompletely understood. However, despite promising preclinical findings, major translational challenges remain, largely because conventional experimental models incompletely reproduce the biological complexity and heterogeneity of human epilepsy.

Emerging experimental platforms may therefore facilitate the integration of this expanded mechanistic understanding into therapeutic development and evaluation. In this review, we summarize current molecular therapeutics and emerging network-oriented approaches in epilepsy, and discuss how next-generation experimental platforms may facilitate the development of more precise and mechanism-based therapies.

This narrative review was informed by literature searches conducted primarily in PubMed and Google Scholar for articles published between 2015 and January 2026. Earlier landmark studies were also included when necessary to provide historical background and mechanistic context. Search terms included combinations of “epilepsy,” “epileptogenesis,” “drug-resistant epilepsy,” “antiseizure therapy,” “precision medicine,” “neuroinflammation,” “network neuroscience,” “brain organoids,” “organ-on-a-chip,” “artificial intelligence,” “induced pluripotent stem cells,” “multielectrode arrays,” “multi-omics,” and related terms. Studies were selected according to their relevance to the scope of the review, methodological rigor, mechanistic significance, translational or clinical relevance, and recency. Priority was given to recent, high-quality original studies and authoritative review articles published in peer-reviewed journals, while seminal publications were included when they provided an essential conceptual foundation. Targeted searches were subsequently conducted through July 2026 to update information on emerging therapeutic pipelines. Clinical trial phases, recruitment status, and registry identifiers were verified using ClinicalTrials.gov through July 2026.

## 2. Multiscale Pathophysiology of Epilepsy

### 2.1. Epileptogenesis as a Network Transformation

Epilepsy is no longer viewed as a disorder arising from a single focal lesion or isolated cellular abnormality, but rather as a dynamic process involving progressive reorganization of neural circuits across multiple spatial and temporal scales. The concept of epileptogenesis captures this transition from a normal brain to a chronically hyperexcitable network capable of generating spontaneous recurrent seizures [12,13].

This process is typically initiated by a diverse range of insults—including genetic mutations, traumatic brain injury, infection, or prolonged seizures—that trigger a cascade of molecular, cellular, and network-level changes. Importantly, these changes do not immediately manifest as epilepsy; instead, they unfold during a latent period characterized by ongoing circuit remodeling. Over time, this remodeling leads to the emergence of pathological network dynamics, including hypersynchrony and aberrant oscillatory activity, which underlie seizure generation and propagation [14,15].

**Figure 1:**
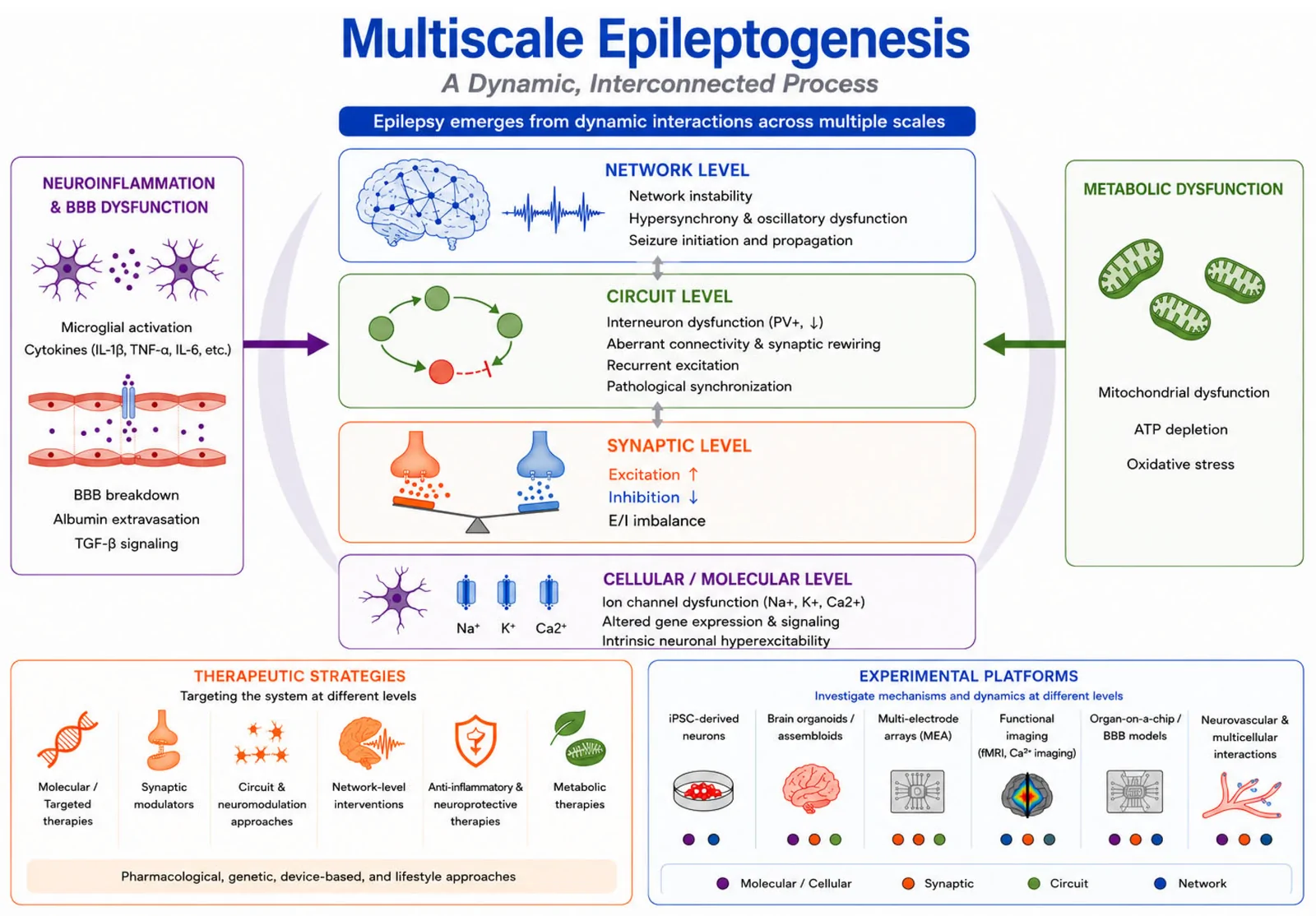
Multiscale framework of epileptogenesis and its implications for therapeutic intervention and experimental modeling. Molecular, cellular, synaptic, inflammatory, metabolic, and neurovascular alterations interact to reshape circuit dynamics and large-scale networks. Recurrent seizures further reinforce pathological network states. Therapeutic strategies act at different levels, whereas complementary experimental platforms help evaluate their effects across interacting biological scales.

From this perspective, epilepsy is best conceptualized as a disorder of network instability, where the balance between excitation and inhibition is not simply shifted, but dynamically reconfigured [15,16]. This network-centric view provides a unifying framework that integrates findings across different levels of analysis and highlights the importance of studying epilepsy as a multiscale phenomenon (Figure 1) [17,18].

### 2.2. Circuit-Level Dysregulation and Hypersynchrony

At the core of epileptic activity lies the emergence of hypersynchronous neuronal firing, reflecting a breakdown in the mechanisms that normally constrain coordinated activity within neural circuits. While an imbalance between excitatory and inhibitory signaling has long been recognized as a central feature of epilepsy, recent work emphasizes that this imbalance is highly circuit-specific and temporally dynamic [15].

In cortical and hippocampal networks, inhibitory interneurons—particularly parvalbumin- and somatostatin-expressing subtypes—play critical roles in regulating the timing and synchronization of pyramidal neuron activity [16]. Dysfunction or loss of these interneurons can lead to impaired feedforward and feedback inhibition, thereby facilitating the emergence of synchronized population discharges. However, inhibition is not uniformly reduced in all epileptic states; in some contexts, aberrant or mistimed inhibitory activity may paradoxically contribute to synchronization and seizure initiation [15,16].

A distinct example of circuit-level dysregulation is observed in absence seizures, in which pathological oscillations emerge through reciprocal interactions within corticothalamic networks. Rather than originating from a localized epileptogenic focus, absence seizures arise from abnormal synchronization between cortical and thalamic circuits, giving rise to the characteristic generalized spike-and-wave discharges. This illustrates that circuit-level dysfunction in epilepsy is syndrome-specific and may emerge through distinct patterns of pathological network synchronization [19].

In parallel, excitatory circuits undergo structural and functional reorganization that promotes recurrent excitation. For example, aberrant axonal sprouting, such as mossy fiber sprouting in the hippocampus, can create new excitatory loops that amplify network activity [12]. These changes are often accompanied by alterations in synaptic strength and plasticity, leading to a state in which normal mechanisms of learning and adaptation are co-opted to support pathological activity.

Importantly, these circuit-level alterations are reflected in changes in network oscillations. Epileptic networks often exhibit abnormal rhythmic activity across multiple frequency bands, as well as altered cross-frequency coupling [20,21]. Such oscillatory disruptions not only serve as biomarkers of network dysfunction but also provide mechanistic insight into how seizures emerge from ongoing brain activity.

### 2.3. Cellular and Synaptic Mechanisms

At the cellular level, epileptogenesis involves a complex interplay between intrinsic neuronal properties and synaptic function. Changes in ion channel expression and function—commonly referred to as channelopathies—can directly alter neuronal excitability [22]. Mutations in voltage-gated sodium, potassium, and calcium channels have been implicated in a range of genetic epilepsies, while activity-dependent modulation of these channels contributes to acquired forms of the disorder [23].

Beyond intrinsic excitability, synaptic alterations play a crucial role in shaping network behavior. Long-term potentiation (LTP) and long-term depression (LTD), which are essential for normal synaptic plasticity, may become dysregulated in epileptic circuits [24]. This can result in the stabilization of hyperexcitable pathways and the weakening of inhibitory control. In this sense, epileptogenesis can be viewed as a form of maladaptive plasticity, where mechanisms that normally support learning are redirected toward the reinforcement of pathological network states [23,24].

Another important feature is the emergence of recurrent connectivity patterns that are not present in healthy circuits. Structural changes such as axonal sprouting and dendritic remodeling contribute to the formation of these aberrant networks [23]. Together, these cellular and synaptic changes provide the substrate upon which large-scale network dysfunction develops.

### 2.4. Neuroinflammation and Glial Contributions

In recent years, it has become increasingly clear that epilepsy is not solely a neuronal disorder, but also involves significant contributions from glial cells and inflammatory processes [11,25]. Following an initial insult, activation of microglia and astrocytes leads to the release of pro-inflammatory cytokines, including IL-1β and TNF-α, which can modulate neuronal excitability and synaptic transmission [25,26].

Astrocytes, in particular, play a key role in maintaining extracellular ion balance and neurotransmitter homeostasis. Disruption of astrocytic functions—such as impaired potassium buffering or glutamate uptake—can create a microenvironment that favors hyperexcitability [27]. Additionally, alterations in astrocyte-neuron signaling may contribute to the synchronization of neuronal activity across networks.

Breakdown of the blood–brain barrier (BBB) represents another important mechanism linking inflammation to epileptogenesis. Leakage of serum proteins, such as albumin, into the brain parenchyma can activate signaling pathways that promote excitatory synaptogenesis and network instability [28,29]. These findings support the notion that neuroinflammation is not merely a consequence of seizures, but an active driver of disease progression [26].

### 2.5. Metabolic and Energetic Dysregulation

Neuronal activity is tightly coupled to energy metabolism, and disruptions in metabolic homeostasis can significantly impact network function. In epilepsy, mitochondrial dysfunction and oxidative stress have been widely reported, leading to impaired ATP production and increased vulnerability to excitotoxic damage [30,31].

Alterations in glucose metabolism and lactate dynamics may further contribute to network instability. Given the high energetic demands of synchronized neuronal firing, even subtle deficits in energy supply can exacerbate pathological activity [31]. These metabolic considerations are particularly relevant in light of therapeutic approaches such as ketogenic diets, which aim to modulate brain metabolism to reduce seizure susceptibility [32].

### 2.6. Epilepsy as a Multiscale Network Disorder

Taken together, these findings support a unified view of epilepsy as a multiscale network disorder (Figure 1). Molecular and cellular changes do not operate in isolation, but interact to reshape circuit dynamics and large-scale brain networks [17,18]. The transition to epilepsy involves not only increased excitability, but also altered patterns of connectivity, synchronization, and information flow [17,33,34].

This perspective has important implications for both research and therapeutic development. Rather than suggesting that effective therapies must directly target every biological scale, it emphasizes the importance of understanding how interventions acting at specific molecular, cellular, or circuit levels influence the broader epileptogenic network. Moreover, it underscores the need for experimental approaches capable of capturing these multiscale interactions, thereby facilitating the translation of mechanistic insights into therapeutic development, evaluation, and clinical application [18].

## 3. Current Molecular Therapeutics and Emerging Network-Level Approaches in Epilepsy

Epilepsy is a complex neurological disorder characterized by diverse pathophysiological mechanisms, including ion channel dysfunction, synaptic imbalance, and network-level abnormalities [35,36]. The therapeutic landscape for epilepsy has evolved considerably, extending beyond conventional antiseizure medications toward increasingly diverse and targeted treatment strategies [37,38].

Recent therapeutic advances include agents that modulate multiple molecular targets, disease-specific therapies directed at signaling pathways such as mTOR and neuroinflammatory cascades, and emerging approaches aimed at restoring ionic homeostasis and metabolic dysfunction [7,39,40,41]. In addition, neuromodulation, gene-based therapies, and other precision medicine approaches continue to broaden the range of available therapeutic options [37,38]. This section reviews current and emerging therapeutic strategies for epilepsy and highlights recent progress across these diverse therapeutic modalities.

### 3.1. Molecular Regulation of Neuronal Excitability

#### 3.1.1. Sodium and Potassium Channel Modulation

Voltage-gated sodium (Na_V_) channel inhibitors and Kv7 potassium channel openers remain major strategies for controlling intrinsic neuronal excitability. Established Na_V_-targeting ASMs suppress repetitive firing, whereas emerging Kv7 modulators such as XEN1101, BHV-7000, and ETX-123 enhance M-currents and membrane stabilization [42,43,44,45].

Together, these interventions directly address excitation–inhibition (E/I) imbalance, one of the central pathophysiological features of epileptogenesis [36,46]. Increasing evidence further suggests that their effects extend beyond simple suppression of individual neuronal firing. By reducing excessive excitatory drive and recurrent activity, sodium and potassium channel modulators indirectly attenuate hypersynchronous network activity and seizure propagation within epileptic circuits [44,47].

Recent developments increasingly emphasize subtype-selective and precision-based regulation of pathological excitability [48,49]. Nevertheless, the long-term effects of ion-channel modulation on circuit remodeling and network organization remain incompletely understood.

#### 3.1.2. Calcium-Dependent Synaptic Excitability

Calcium-dependent synaptic transmission plays a central role in regulating neuronal excitability and seizure propagation in epilepsy. Several ASMs, including gabapentin and pregabalin, reduce excitatory neurotransmitter release through binding to the α_2_δ subunit of voltage-gated calcium channels at presynaptic terminals, thereby limiting calcium influx and glutamate release [50,51]. In addition, ethosuximide suppresses T-type Ca^2+^ channel activity associated with thalamocortical burst firing in absence seizures, while agents such as zonisamide and valproate exert partial modulatory effects on calcium currents [52,53,54].

Unlike sodium and potassium channel modulators that primarily regulate intrinsic membrane excitability, calcium channel–targeting therapies exert many of their effects through modulation of synaptic transmission and presynaptic neurotransmitter release [55]. Altered Ca^2+^ channel activity contributes to enhanced glutamatergic signaling, excessive synaptic excitation, and recurrent network activity in epileptic circuits. By reducing calcium-dependent excitatory transmission, these therapies indirectly attenuate hypersynchronous firing and seizure propagation, thereby contributing to stabilization of hyperexcitable networks [56,57,58].

Recent therapeutic development has increasingly focused on subtype-selective modulation of calcium signaling and presynaptic release dynamics [59,60]. These approaches aim to improve therapeutic precision while minimizing off-target effects associated with broad calcium channel inhibition. These developments highlight the growing importance of presynaptic and calcium-dependent regulation in controlling excitatory network activity.

#### 3.1.3. Excitatory–Inhibitory Synaptic Balance

Disruption of excitatory–inhibitory (E/I) synaptic balance is a central mechanism underlying epileptogenesis and pathological network synchronization. Pharmacological strategies targeting GABAergic inhibition and glutamatergic excitation therefore represent major approaches for restoring synaptic stability in epilepsy [61,62]. Clinically established GABA_A_-targeting agents—including benzodiazepines, barbiturates, stiripentol, and ganaxolone—enhance inhibitory neurotransmission through positive allosteric modulation of GABA_A_ receptors, thereby increasing chloride influx and suppressing excessive neuronal firing [63]. In contrast, glutamatergic modulators such as perampanel, topiramate, and felbamate reduce excitatory transmission through AMPA and NMDA receptor inhibition [64].

Although these therapies operate through distinct receptor systems, both ultimately function to reduce pathological excitation and stabilize hyperexcitable circuits. Enhancement of inhibitory signaling suppresses recurrent firing and network hypersynchrony, whereas inhibition of glutamatergic transmission attenuates excitatory propagation and maladaptive synaptic activity [57,61]. Increasing evidence further suggests that these agents influence not only acute seizure activity, but also pathological oscillations and abnormal synchronization within epileptogenic networks [57].

Next-generation GABAergic compounds aim to improve tolerability and minimize sedation, while emerging glutamatergic therapies—including selective NMDA receptor subtype modulators and mGluR-targeting agents—seek to regulate excitatory network dysfunction more precisely [63]. Emerging receptor-selective therapies increasingly aim to regulate pathological synaptic plasticity and abnormal oscillatory synchronization with greater precision, thereby linking synaptic modulation to broader circuit-level stabilization [65].

### 3.2. Circuit and Network-Level Modulation

#### 3.2.1. Circuit Stabilization and Network Synchronization

Increasing evidence suggests that epilepsy cannot be fully understood solely as a disorder of intrinsic neuronal hyperexcitability, but rather as a disease of pathological circuit synchronization and maladaptive network dynamics. In particular, recurrent excitation, impaired inhibitory control, abnormal oscillatory activity, and hypersynchronous firing collectively contribute to seizure generation and propagation across distributed neural circuits [66]. High-frequency oscillations (HFOs) and aberrant synchronization patterns are increasingly recognized as functional markers of epileptogenic networks [67].

Several ASMs appear to exert circuit-level effects by modulating synaptic transmission and network synchronization. SV2A-targeting agents such as levetiracetam and brivaracetam regulate synaptic vesicle release and reduce excessive glutamatergic transmission, thereby attenuating recurrent excitatory activity and hypersynchronous firing [68]. Experimental evidence further suggests that levetiracetam may partially restore abnormal CA3–CA1 synaptic transmission and short-term synaptic plasticity, indicating potential stabilizing effects on epileptogenic circuits beyond simple seizure suppression [69].

Restoration of inhibitory circuit function also represents an important mechanism of network stabilization. Dysfunction of parvalbumin- and somatostatin-expressing interneurons contributes to impaired inhibitory synchronization and pathological oscillatory activity in epilepsy [70,71]. GABAergic therapies, including benzodiazepines and ganaxolone, indirectly enhance inhibitory circuit stability, whereas emerging precision therapies such as STK-001 aim to restore interneuron function more directly in sodium voltage-gated channel alpha subunit 1 (SCN1A)-related epilepsies [72,73]. In parallel, serotonergic neuromodulation through agents such as fenfluramine and LP352 may influence large-scale circuit excitability and oscillatory synchronization through modulation of distributed neuromodulatory systems [74,75].

These approaches reflect a gradual transition from suppression of individual neuronal firing toward modulation of pathological network dynamics and synchronization states. Nevertheless, direct pharmacological regulation of large-scale oscillatory activity, maladaptive circuit remodeling, and long-term epileptogenic network organization remains comparatively underdeveloped relative to molecular and receptor-level targeting [76]. A conceptual summary of how currently established ASMs may influence synaptic plasticity and network-level dynamics beyond their primary molecular targets is presented in Table 1.

#### 3.2.2. Neuromodulatory Circuit Regulation

Beyond direct modulation of excitatory and inhibitory synaptic transmission, increasing attention has focused on neuromodulatory systems that regulate large-scale circuit excitability and network synchronization. Among these, serotonergic signaling has emerged as an important mechanism influencing seizure susceptibility, oscillatory activity, and pathological synchronization, particularly in developmental and epileptic encephalopathies (DEEs) [77]. Unlike classical receptor-targeting ASMs, neuromodulatory therapies are thought to influence distributed network dynamics through broader regulation of circuit state and excitability [78,79].

Fenfluramine represents a representative example of serotonergic neuromodulation in epilepsy. Its antiseizure effects involve enhanced serotonin release together with agonistic activity at multiple serotonin (5-hydroxytryptamine, 5-HT) receptor subtypes, including 5-HT_1D_ and 5-HT_2C_ receptors [80]. Experimental and clinical evidence suggests that serotonergic signaling can influence excitatory–inhibitory balance, oscillatory synchronization, and large-scale circuit stability, thereby attenuating hypersynchronous network activity associated with seizure generation and propagation [77,81].

Growing attention has been directed toward receptor subtype–specific regulation of neural circuits. LP352, a selective 5-HT_2C_ receptor superagonist, exemplifies this transition by aiming to preserve antiseizure efficacy while minimizing off-target serotonergic effects [82]. These approaches reflect a broader movement toward precision neuromodulation, where therapies increasingly target circuit-level regulatory systems rather than individual molecular determinants of excitability alone [76,83].

Although neuromodulatory approaches provide a potentially important bridge between molecular pharmacology and systems-level network regulation, the mechanisms linking neuromodulatory signaling to long-term circuit remodeling and epileptogenic network organization remain incompletely understood [76].

### 3.3. Neuroinflammatory and Neurovascular Modulation

Neuroinflammatory and neurovascular dysfunction have emerged as major contributors to epileptogenesis and chronic network instability [9,25]. Following brain injury or recurrent seizures, activation of microglia and astrocytes leads to the release of pro-inflammatory cytokines, including IL-1β and TNF-α, which can enhance neuronal excitability, disrupt synaptic transmission, and promote pathological synchronization across epileptic networks [25,84]. In parallel, breakdown of the blood–brain barrier (BBB) permits serum proteins and inflammatory mediators to enter the brain parenchyma, further amplifying glial activation, excitatory synaptogenesis, and maladaptive circuit remodeling [85]. Increasing evidence therefore suggests that neuroinflammation and BBB dysfunction are not merely secondary consequences of seizures, but active drivers of epileptogenic network reorganization.

Disease-modifying therapeutic strategies targeting these pathways aim to interrupt upstream mechanisms of epileptogenesis rather than simply suppress acute seizure activity [86]. VX-765, a caspase-1 inhibitor, reduces IL-1β–mediated inflammatory signaling implicated in seizure generation and network hyperexcitability [87]. Cannabidiol additionally exhibits anti-inflammatory and neuromodulatory effects that may influence glial activation and epileptogenic network dysfunction [88,89]. More broadly, anti-inflammatory therapies, astrocytic modulators, and approaches designed to restore ionic and metabolic homeostasis may collectively contribute to stabilization of neurovascular and inflammatory network disturbances [86,90].

These developments increasingly support a transition from neuron-centered models of epilepsy toward integrated neuro-glial-vascular frameworks in which epileptogenesis emerges through interactions among neurons, glial cells, inflammatory signaling, and vascular dysfunction across multiple biological scales [91,92]. Emerging experimental systems, including BBB-integrated organ-on-a-chip platforms, are further enabling investigation of how neurovascular dysfunction contributes to pathological network synchronization and therapeutic response [93,94].

### 3.4. Precision Restoration of Epileptogenic Networks

Recent advances in molecular genetics and gene-targeting technologies have accelerated the development of precision therapeutics aimed at restoring specific pathological components of epileptogenic networks [49,95,96]. Unlike conventional ASMs, which primarily suppress neuronal excitability or synaptic transmission, these approaches seek to correct disease-causing molecular abnormalities that disrupt circuit stability and network organization [95,97]. This transition reflects an emerging view of epilepsy as a disorder arising from dysfunction of identifiable cellular, synaptic, and network components rather than from generalized hyperexcitability alone.

One major strategy involves restoration of ion channel and inhibitory interneuron function [98]. In SCN1A-related epilepsies such as Dravet syndrome, loss-of-function mutations impair Na_V_1.1 activity predominantly in inhibitory interneurons, leading to severe excitation–inhibition (E/I) imbalance and hypersynchronous network activity [99]. STK-001, an antisense oligonucleotide (ASO) therapy designed to increase SCN1A expression, aims to restore interneuron excitability and normalize pathological circuit activity rather than simply suppress seizures symptomatically [100].

Additional precision approaches target excitatory circuit dysfunction and maladaptive plasticity more directly. AMT-260 utilizes AAV-mediated microRNA delivery to suppress glutamate ionotropic receptor kainate type subunit 2 (GRIK2) expression within epileptogenic regions, thereby reducing kainate receptor-mediated recurrent excitation and pathological synchronization [101,102]. Emerging RNA-based therapies such as NMT.001 further extend this concept by modulating microRNA-134 (miR-134)-associated pathways involved in dendritic remodeling, excitatory plasticity, and maladaptive network reorganization [103,104].

Collectively, current and emerging therapeutic strategies have substantially broadened the treatment landscape of epilepsy. Representative approved and emerging therapeutic pipelines, together with their regulatory/clinical status and key indications, are summarized in Table 2. These strategies target not only ion channels and synaptic transmission but also circuit synchronization, neuromodulatory systems, neuroinflammatory pathways, and disease-specific molecular abnormalities.

Despite these advances, CE remains a multifactorial problem that cannot be attributed to the failure of a single molecular target. Alterations in therapeutic targets, drug transport and distribution, disease heterogeneity, and progressive network reorganization may interact to shape treatment response [1,7]. Consequently, acute seizure suppression alone may not predict sustained clinical benefit, and the effects of molecular, synaptic, or circuit-level interventions on long-term network remodeling and neuro-glial interactions remain incompletely understood [1,91,92,105]. At the clinical level, molecular and circuit-level biomarkers need to be interpreted alongside functional measures such as EEG and network signatures and long-term seizure outcomes to support patient selection and treatment-response prediction. AI-based prediction of ASM responsiveness and the association between long-term network reorganization and responsive neurostimulation outcomes illustrate this emerging direction [6,48,105]. These challenges are compounded by conventional experimental models, which often fail to capture human cellular heterogeneity, developmental trajectories, and multiscale network responses to therapy [106,107]. Consequently, next-generation experimental platforms capable of integrating patient-specific molecular phenotypes, functional network readouts, and neurovascular analyses may help connect mechanistic insights with therapeutic development, evaluation, and clinical translation [108,109].

## 4. Emerging Experimental Platforms for Epilepsy Research and Therapeutic Development

To address these translational challenges, the field has increasingly turned toward emerging experimental platforms, including New Approach Methodologies (NAMs), that provide more physiologically relevant, human-specific, and integrative frameworks for investigating disease mechanisms and therapeutic responses across biological scales [109,110]. This section reviews conventional experimental approaches and the complementary roles of next-generation technologies in epilepsy research and therapeutic development.

### 4.1. Conventional Experimental Approaches

Conventional two-dimensional neuronal cultures have been valuable for dissecting ion-channel, neurotransmitter, and neuron–glia mechanisms and for early pharmacological screening [111,112]. However, their planar architecture, limited cellular diversity, and immature phenotypes restrict their ability to model the tissue organization and network interactions central to human epilepsy [113,114].

**Figure 2:**
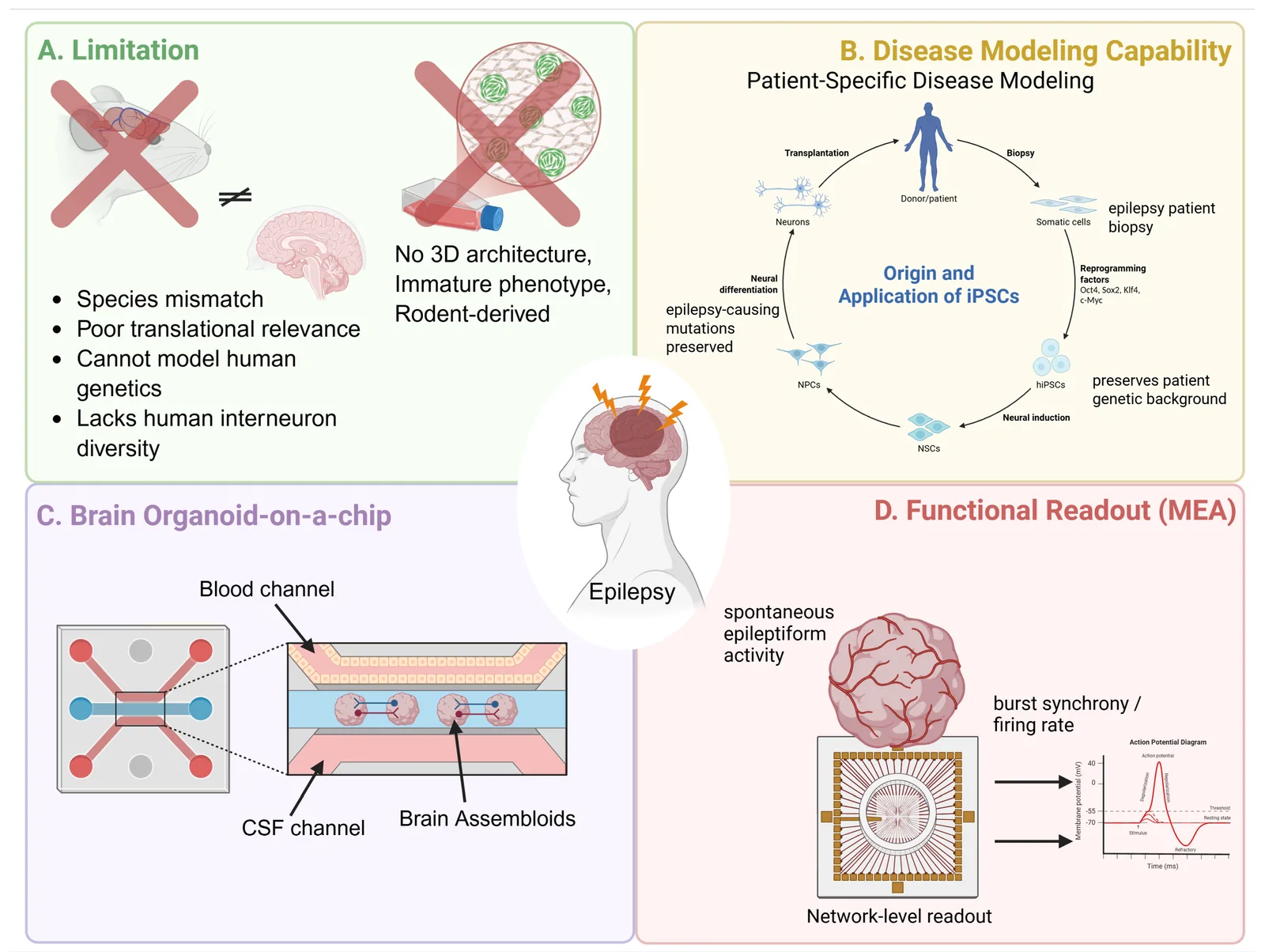
Emerging Experimental Platforms for Human-Relevant Epilepsy Modeling and Therapeutic Evaluation. (**A**) Limitations of conventional animal and 2D models. (**B**) Patient-specific iPSC-derived neural models. (**C**) Brain organoid-on-a-chip systems integrating interregional and neurovascular components. (**D**) MEA-based functional readouts of epileptiform activity and drug response. Created in BioRender (https://BioRender.com/02qaiya (accessed on 19 June 2026)).

Animal models—including chemoconvulsant, kindling, and genetic paradigms—have been indispensable for studying seizure propagation, neuronal injury, synaptic plasticity, behavioral comorbidities, and ASM discovery [106,115]. Their translational value is nevertheless limited by species differences, variability in experimental protocols, and weaker prediction of disease-modifying efficacy or pharmacoresistant epilepsy [107,116,117]. These limitations support the complementary use of human-based experimental platforms (Figure 2).

### 4.2. New Approach Methodologies (NAMs)

Recent technological advances have enabled the emergence of NAMs designed to better capture human neurobiology and the complex cellular environments underlying epilepsy. These approaches integrate stem cell biology, bioengineering, high-resolution electrophysiology, multi-omics technologies, and computational modeling to create more physiologically relevant systems for studying epileptic mechanisms.

Among these advances, human induced pluripotent stem cell (iPSC) technology has transformed the landscape of disease modeling. By reprogramming somatic cells such as fibroblasts into pluripotent stem cells, researchers can generate patient-specific neural cell types carrying the exact genetic background of individuals with epilepsy [114]. iPSC-derived neurons have been widely used to study monogenic epileptic disorders, including channelopathies associated with mutations in genes such as SCN1A, KCNQ2, and SCN2A [114]. These models enable direct investigation of disease-relevant electrophysiological phenotypes, including altered sodium channel activity, disrupted potassium currents, and imbalances between excitatory and inhibitory neuronal signaling [118]. The primary advantage of iPSC-based models lies in their ability to preserve patient-specific genetic variability, thereby providing a platform for personalized disease modeling and drug testing [119]. Additionally, genome editing technologies such as CRISPR/Cas9 allow the generation of isogenic control lines in which specific mutations are corrected or introduced, facilitating causal investigation of genetic variants [114]. iPSC technology provides a crucial human cellular foundation upon which more complex experimental systems are built.

Building upon stem cell technology, brain organoids and assembloids have emerged as powerful three-dimensional models that recapitulate aspects of human brain development and organization. Brain organoids are self-organizing neural tissues derived from pluripotent stem cells that develop spatially structured progenitor zones and neuronal layers reminiscent of early cortical development. Over time, these structures generate diverse neuronal and glial populations and establish spontaneous neuronal activity [120,121]. In the context of epilepsy research, organoid models have been particularly informative for studying developmental and epileptic encephalopathies [121]. Patient-derived organoids have reproduced disease phenotypes associated with disorders such as Rett syndrome, Timothy syndrome, and WWOX-related epileptic encephalopathy, including aberrant neuronal differentiation, disrupted synaptic signaling, and hypersynchronous network activity [117].

To rigorously model epileptic phenotypes in vitro, researchers utilize two primary strategies: capturing intrinsic genetic susceptibility from patient-derived iPSCs or chemically inducing hypersynchronous states in healthy organoids [122]. In genetic models, patient-specific configurations preserve native mutational loads, naturally giving rise to cellular hyperexcitability and spontaneous seizure-like bursting patterns [119]. Alternatively, acute seizure-in-a-dish states can be robustly triggered in wild-type or mature organoids through chemical exposure—such as administration of the chemoconvulsant kainic acid or exposure to low-magnesium/high-potassium artificial cerebrospinal fluid (aCSF) [123,124,125]. These chemical challenges impair baseline inhibitory homeostatic boundaries, co-opting normal physiological pathways into synchronized population bursts that effectively mimic the electrographic benchmarks of acute clinical seizures [126].

More recently, the development of assembloid systems—generated by fusing regionally specified organoids—has enabled modeling of interregional neural interactions such as interneuron migration from the ganglionic eminence to the cortex [127]. These systems provide a unique opportunity to investigate how developmental disturbances in neuronal migration or circuit formation contribute to epileptic pathology [123]. Beyond modeling developmental abnormalities, organoid and assembloid systems can be used to examine how disease-associated molecular and cellular defects become expressed as abnormalities in circuit formation and network activity. Because developmental, cellular, and electrophysiological phenotypes can be assessed within the same human-derived system, these models provide a means to connect mechanisms operating across multiple biological scales [120,121,123,127]. These integrated platforms also provide a framework for evaluating the mechanism-based precision therapies discussed in Chapter 3. For example, SCN1A-directed ASOs such as STK-001 could be evaluated in patient-derived assembloids by examining GABAergic interneuron phenotypes and synchronized network activity. Gene-replacement and microRNA-based approaches could likewise be assessed for their effects on maladaptive synaptic and circuit remodeling [72,101,102,103,123,127,128].

Functional characterization of neuronal networks within these emerging models has been greatly facilitated by advances in electrophysiological technologies, particularly multi-electrode arrays (MEAs) [129]. MEAs consist of grids of microelectrodes embedded in culture substrates that enable simultaneous recording of extracellular electrical activity from multiple neurons [130]. Unlike traditional patch-clamp electrophysiology, which measures activity at the single-cell level, MEAs allow long-term monitoring of network-level dynamics across large neuronal populations [111]. This capability is particularly valuable for epilepsy research, as seizures represent emergent phenomena arising from coordinated neuronal activity [131]. MEA platforms can quantify firing rates, bursting patterns, synchronization indices, and epileptiform discharges, providing a functional readout of network excitability in response to genetic mutations or pharmacological perturbations [112]. These network-level readouts make MEAs particularly useful for determining whether interventions acting at molecular or cellular levels ultimately alter pathological bursting and synchronization [112,131]. Technological advances have further expanded the capabilities of MEA systems. High-density complementary metal–oxide–semiconductor (CMOS) arrays now permit recordings from thousands of electrodes simultaneously, enabling high-resolution mapping of neuronal activity [132]. Three-dimensional electrode meshes and flexible probes have also been developed to interface with organoids and other 3D tissue constructs [133,134]. MEAs have become a central component of modern in vitro seizure modeling and high-throughput drug screening.

Complementing these advances in cellular modeling and electrophysiology, microengineering technologies have enabled the development of organ-on-a-chip platforms and microphysiological systems that recreate key aspects of tissue microenvironments [135]. These systems employ microfluidic channels and compartmentalized culture chambers to control fluid flow, mechanical forces, and biochemical gradients, thereby reproducing physiological conditions more accurately than static culture systems [136]. In the context of epilepsy research, neural microphysiological platforms can be designed to mimic specific circuit architectures or to investigate interactions between neuronal networks and the blood–brain barrier (BBB) [137,138]. For example, microfluidic devices connecting distinct neuronal populations through microtunnels allow the study of seizure propagation across defined pathways [135], while integrated BBB models enable investigation of neurovascular interactions and drug permeability [139,140].

One of the key advantages of organ-on-a-chip technologies is their ability to incorporate multiple cell types—including neurons, astrocytes, microglia, and endothelial cells—within controlled microenvironments [141]. This multi-lineage capability bridges a crucial gap regarding the neuroinflammatory and neurovascular drivers highlighted in Chapter 2.4 and Chapter 3.3. By engineering a “Blood–Brain Barrier (BBB)-on-a-chip,” investigators can simulate physiological fluid shear stresses and monitor real-time interactions between brain endothelial cells and perivascular astrocytes [142]. This allows for the precise, mechanistic dissection of how BBB breakdown, systemic albumin leakage, and the subsequent release of pro-inflammatory cytokines (such as IL-1β and TNF-α) drive hypersynchronous circuit dynamics [140]. Therapeutic strategies discussed in Section 3.3, such as the caspase-1 inhibitor VX-765 or astrocyte-targeted interventions, could be evaluated in these systems to determine whether they attenuate upstream inflammatory signaling, neurovascular dysfunction, and associated changes in network activity [138,140,143].

Parallel to advances in experimental modeling, high-throughput molecular profiling technologies have provided unprecedented insights into the molecular landscape of epilepsy [144]. Multi-omics approaches—including transcriptomics, epigenomics, proteomics, metabolomics, and spatially resolved omics—enable comprehensive characterization of cellular states and signaling pathways involved in epileptic networks [145]. Single-cell RNA sequencing, for instance, has revealed diverse neuronal and glial subpopulations within epileptic tissue and identified transcriptional signatures associated with neuroinflammation, synaptic remodeling, and metabolic dysfunction [146]. Spatial transcriptomics technologies further extend these analyses by preserving spatial information, allowing researchers to map molecular changes across specific brain regions involved in seizure generation [147].

Integrating diverse molecular layers requires computational frameworks capable of handling high-dimensional data and identifying biologically meaningful patterns [148]. Artificial intelligence (AI) and machine learning approaches have therefore become increasingly important in epilepsy research [149]. AI-based algorithms can integrate multimodal datasets—including electrophysiological recordings, genomic profiles, and neuroimaging data—to uncover previously unrecognized relationships and predict disease outcomes [150,151]. In experimental systems, machine learning models are used to analyze large-scale MEA recordings, classify seizure-like events, and identify drug-responsive phenotypes [131,149,152]. Together, multi-omics and AI-based analyses can link molecular states with electrophysiological and network phenotypes across experimental platforms [144,149,152].

### 4.3. Toward an Integrated Experimental Framework

Collectively, emerging experimental platforms are reshaping the study of epilepsy by enabling investigation across multiple biological scales—from molecular mechanisms to neuronal networks and tissue-level interactions [109]. Whereas traditional models have largely relied on reductionist approaches, modern technologies emphasize integration and human relevance [106]. The convergence of stem cell–derived neural systems, advanced electrophysiological monitoring, microengineered tissue environments, multi-omics profiling, and computational analysis offers an unprecedented opportunity to construct more predictive models of epileptogenesis.

Despite their potential, current NAMs retain platform-specific methodological and technical constraints. iPSC-derived neurons often remain developmentally immature and vary across cell lines and differentiation batches, whereas organoid models show incomplete maturation, batch-to-batch variability, and limited vascularization [114,153,154]. MEA studies require advanced analysis of high-dimensional electrophysiological recordings, organ-on-a-chip systems remain difficult to standardize and scale, and multi-omics and AI-based approaches depend on representative datasets and interpretable analytical frameworks [148,155,156,157,158,159,160]. Current organoid and microfluidic systems also mainly reproduce localized circuits and do not yet fully model long-range seizure propagation across distributed brain regions [123]. These limitations may be partially addressed through the complementary use of experimental platforms, including multiregional assembloids and interconnected organ-on-a-chip systems that extend current models toward interregional interactions and long-range propagation [161,162].

Building on these complementary approaches, future research will focus on combining these technologies into unified experimental pipelines. For example, patient-derived iPSC lines can be used to generate brain organoids that are integrated into microfluidic organ-on-a-chip systems incorporating vascular and immune components [141,142,154]. Network activity within these systems can be monitored using high-density MEAs [132,133,134], while parallel multi-omics analyses provide molecular insight into cellular responses [144,145,147,163]. AI-driven computational models can then integrate these diverse datasets to identify mechanistic pathways and predict therapeutic responses [149,152]. Such integrated experimental frameworks have the potential to transform the discovery and evaluation of antiseizure therapies. By capturing patient-specific variability, modeling complex neurovascular and neuroimmune interactions, and providing high-resolution functional and molecular readouts, these platforms may significantly improve the predictive power of preclinical studies [152]. Ultimately, the continued development and integration of these emerging technologies may pave the way toward precision medicine approaches for epilepsy, enabling more effective and individualized treatment strategies for patients who remain resistant to current therapies.

**Table 1:** Network and synaptic plasticity perspectives on antiseizure medications.

Drug Class/Agent	Primary Target(s)	Synaptic Plasticity Perspective	Network-Level Perspective
Lamotrigine	Voltage-gated Na^+^ channels, glutamate release	Tends to suppress excessive LTP-like plasticity [164]	Reduces propagation of hyperexcitable activity [165]
Valproate	GABAergic signaling, Na^+^ channels, HDAC inhibition	Dual role: restoration of synaptic plasticity versus developmental neurotoxicity [166]	May suppress pathological network remodeling [166]
Levetiracetam	SV2A	Partial restoration of synaptic transmission and plasticity [69]	Reduces seizure-related synchronization [167]
Carbamazepine/Oxcarbazepine	Voltage-gated Na^+^ channels	Indirect normalization of aberrant synaptic plasticity [168]	Suppresses seizure propagation [168]
Perampanel	AMPA receptors	Inhibits excitatory synaptic transmission [169]	Presumed reduction in HFOs and pathological synchronization [169]
Brivaracetam	SV2A	Regulates synaptic vesicle release [170]	May contribute to network stabilization through regulation of synaptic vesicle release [170]
Cenobamate	Voltage-gated Na^+^ channels, GABA_A_ receptors	Suppresses pathological hyperexcitability [171]	Produces strong seizure suppression and network inhibition [171]

Network-level effects represent conceptual interpretations based primarily on preclinical and mechanistic evidence unless otherwise specified.

**Table 2:** Selected approved and emerging antiseizure therapeutic pipelines: mechanisms, regulatory/clinical status, and key indications.

Pipeline	Mechanism/Target	Regulatory/Clinical Stage	Current Status	Key Indications
XEN1101/azetukalner [172,173]	Kv7 (KCNQ) potassium channel opener	Phase 3	Phase 3 completed (NCT05614063)	FOS; PGTCS
BHV-7000/opakalim [174]	Kv7 potassium channel modulator; selective Kv7.2/7.3 activator	Pivotal Phase 2/3	Phase 2/3; Recruiting (NCT06132893); Active, not recruiting (NCT06309966)	Refractory focal epilepsy; KCNQ2-DEE exploratory
OV329 [175]	GABA aminotransferase (GABA-AT) inhibitor	Phase 2 development	Phase 1 completed (No ClinicalTrials.gov record cited); Phase 2 studies planned/initiating	Treatment-resistant FOS, TSC-associated seizures, and infantile spasms
Ganaxolone (Ztalmy) [176]	GABA_A_ receptor positive allosteric modulator (PAM)	Approved for CDD; further clinical development in additional indications discontinued.	Phase 3; Completed (NCT03572933)	CDD-associated seizures
Radiprodil [37]	GluN2B (NR2B)-selective NMDA receptor negative allosteric modulator	Phase 3 in GRIN-NDD; Phase 1/2 in TSC/FCD	Phase 3; Recruiting (NCT07224581); Phase 1/2; Active, not recruiting (NCT06392009)	GRIN-NDD with gain-of-function variants; TSC/FCD type II exploratory
Fenfluramine (Fintepla) [177]	Serotonin release/receptor-based modulation (5-HT_1D_, 5-HT_2_ and sigma-1 actions)	Approved; label and geographic expansion	Approved—no active trial specified	Dravet syndrome and LGS
LP352 (bexicaserin) [178]	Selective 5-HT_2C_ superagonist	Late clinical development	Phase 3; Recruiting (NCT06660394; NCT06719141)	Dravet syndrome, LGS, and other DEEs
STK-001 (zorevunersen)	ASO-mediated SCN1A upregulation/Na_V_1.1 restoration	Phase 3 (EMPEROR)	Phase 3; Recruiting (NCT06872125)	SCN1A loss-of-function Dravet syndrome
AMT-260	AAV9-miRNA gene therapy targeting GRIK2 (GluK2 suppression)	Phase 1/2a clinical development	Phase 1/2a; Recruiting (NCT06063850)	Refractory unilateral mesial temporal lobe epilepsy via GRIK2 knockdown
NMT.001 [37]	miR-134 inhibition strategy (epileptogenic network modulation)	Preclinical	No ClinicalTrials.gov record cited.	Drug-resistant epilepsy
BL-001	Microbiome-based live biotherapeutic product targeting gut–brain axis	Early clinical development	Phase 1a; Completed (NCT05818306)	Dravet syndrome and other DEEs
Rezanecel (NRTX-1001)	Allogeneic human GABAergic inhibitory interneuron transplantation	Phase 1/2 and Phase 3	Phase 3; Recruiting (NCT05135091); Phase 1/2; Recruiting (NCT06422923)	Drug-resistant unilateral and bilateral MTLE

Development status was verified using ClinicalTrials.gov and official sponsor disclosures as of July 2026. CDD, CDKL5 deficiency disorder; FCD, focal cortical dysplasia; FOS, focal onset seizures; GRIN-NDD, GRIN-related neurodevelopmental disorder; LGS, Lennox–Gastaut syndrome; MTLE, mesial temporal lobe epilepsy; PGTCS, primary generalized tonic–clonic seizures.

## 5. Conclusions

In summary, this review reflects a fundamental reconceptualization of epilepsy—from a disorder of neuronal hyperexcitability toward one of multiscale network dysfunction arising from dynamic interactions across molecular, cellular, circuit, neuroinflammatory, and neurovascular levels. Therapeutic development is progressively expanding beyond conventional seizure suppression, with emerging precision strategies targeting disease-specific molecular abnormalities, interneuron dysfunction, neuroinflammatory cascades, and pathological circuit synchronization. However, this expanded understanding of epilepsy pathophysiology has not yet been fully incorporated into therapeutic development and evaluation, and the consequences of therapeutic interventions across interacting biological scales remain incompletely understood. To address this gap, New Approach Methodologies—encompassing iPSC-derived neuronal systems, brain organoids and assembloids, multi-electrode arrays, organ-on-a-chip platforms, multi-omics profiling, and AI-driven computational frameworks—provide complementary, human-relevant tools for linking molecular and cellular mechanisms with circuit, network, neuroinflammatory, and neurovascular phenotypes and for evaluating therapeutic responses across these levels. Rather than replacing established models, the coordinated use of these platforms may help narrow persistent translational gaps and support more predictive disease models and more mechanism-informed therapeutic development and evaluation for patients who remain refractory to current therapies.

## Data Availability

No new data were created or analyzed in this study. Data sharing is not applicable to this article.

## Abbreviations

The following abbreviations are used in this manuscript:

ASM	Antiseizure Medication
ASO	Antisense Oligonucleotide
ATP	Adenosine Triphosphate
AAV	Adeno-Associated Virus
BBB	Blood–Brain Barrier
BHV	Biohaven
Ca^2+^	Calcium Ion
CA3	Cornu Ammonis Area 3
CA1	Cornu Ammonis Area 1
CMOS	Complementary Metal–Oxide–Semiconductor
CNS	Central Nervous System
CRISPR	Clustered Regularly Interspaced Short Palindromic Repeats
CSF	Cerebrospinal Fluid
DEE	Developmental and Epileptic Encephalopathy
E/I	Excitation–Inhibition
EEG	Electroencephalography
GABA	Gamma-Aminobutyric Acid
GABAA	Gamma-Aminobutyric Acid Type A
GRIK2	Glutamate Ionotropic Receptor Kainate Type Subunit 2
HDAC	Histone Deacetylase
HFO	High-Frequency Oscillation
hiPSC	Human Induced Pluripotent Stem Cell
iPSC	Induced Pluripotent Stem Cell
IL-1β	Interleukin-1 Beta
LTD	Long-Term Depression
LTP	Long-Term Potentiation
MEA	Multi-Electrode Array
miRNA	MicroRNA
mGluR	Metabotropic Glutamate Receptor
mTOR	Mammalian Target of Rapamycin
NAM	New Approach Methodology
Na_V_	Voltage-Gated Sodium Channel
NMDA	N-Methyl-D-Aspartate
NPC	Neural Progenitor Cell
NSC	Neural Stem Cell
PAM	Positive Allosteric Modulator
SCN1A	Sodium Voltage-Gated Channel Alpha Subunit 1
STXBP1	Syntaxin Binding Protein 1
SV2A	Synaptic Vesicle Glycoprotein 2A
TNF-α	Tumor Necrosis Factor Alpha
TSC	Tuberous Sclerosis Complex
WWOX	WW Domain-Containing Oxidoreductase
